# Primary Mediastinal and Testicular Germ Cell Tumors in Adolescents and Adults: A Comparison of Genomic Alterations and Clinical Implications

**DOI:** 10.3390/cancers13205223

**Published:** 2021-10-18

**Authors:** Milena Urbini, Giuseppe Schepisi, Sara Bleve, Alessandra Virga, Caterina Gianni, Giorgia Gurioli, Paola Ulivi, Ugo De Giorgi

**Affiliations:** 1 Biosciences Laboratory, IRCCS Istituto Romagnolo per lo Studio dei Tumori (IRST) “Dino Amadori”, Via P. Maroncelli 40, 47014 Meldola, Italy; milena.urbini@irst.emr.it (M.U.); alessandra.virga@irst.emr.it (A.V.); giorgia.gurioli@irst.emr.it (G.G.); 2 Department of Medical Oncology, IRCCS Istituto Romagnolo per lo Studio dei Tumori (IRST) “Dino Amadori”, Via P. Maroncelli 40, 47014 Meldola, Italy; giuseppe.schepisi@irst.emr.it (G.S.); sara.bleve@irst.emr.it (S.B.); caterina.gianni@irst.emr.it (C.G.); ugo.degiorgi@irst.emr.it (U.D.G.)

**Keywords:** mediastinal GCT, molecular, mutation, sequencing, secondary hematologic diseases

## Abstract

**Simple Summary:**

The germ cell tumors (GCTs) family is a heterogeneous group of neoplasms that includes tumors affecting testis (TGCTs) and rarer cases occurring in extragonadal sites. Mediastinal germ cell tumors (MGCTs) are more aggressive and have poorer prognosis. Due to their rarity of MGCTs, few molecular and clinical studies are reported. MGCTs share biological similarities with TGCT, and international guidelines recommend use of the same therapies validated for TGCT. However, while high response rate is achieved in TGCT, MGCT tend to be resistant to therapy. This review resumes all molecular findings reported in MGCTs, summarizing molecular characteristics common with TGCT and highlighting the different molecular alterations that characterize mediastinal tumors. A deeper understanding of the MGCT biology will help in clinical management of these patients.

**Abstract:**

Mediastinal germ cell tumors (MGCTs) share histologic, molecular and biomarkers features with testicular GCTs; however, nonseminomatous MGCTs are usually more aggressive and have poorer prognosis than nonseminomatous TGCTs. Most nonseminomatous MGCT cases show early resistance to platinum-based therapies and seldom have been associated with the onset of one or more concomitant somatic malignancies, in particular myeloid neoplasms with recent findings supporting a common, shared genetic precursor with the primary MGCT. Genomic, transcriptomic and epigenetic features of testicular GCTs have been extensively studied, allowing for the understanding of GCT development and transformation of seminomatous and nonseminomatous histologies. However, MGCTs are still lacking proper multi-omics analysis and only few data are reported in the literature. Understanding of the mechanism involved in the development, in the progression and in their higher resistance to common therapies is still poorly understood. With this review, we aim to collect all molecular findings reported in this rare disease, resuming the similarities and disparities with the gonadal counterparts.

## 1. Introduction

Germ cell tumors (GCTs) are rare neoplasms that originate from progenitor cells of the germ cell lineage. The GCT family is a heterogeneous group of neoplasms, which can affect gonads (ovaries and testes) and in rarer cases (approximately 5%) can occur in extragonadal sites (retroperitoneum, mediastinum, pineal gland). Pineal and mediastinal GCTs are considered primary tumors, while retroperitoneal tumors are suspected to be metastases of a misunderstood gonadal lesion in certain cases [1,2,3].

The most frequent malignant form of GCT affects testis in young men (TGCT), but fortunately it can be considered a highly curable neoplasm. Conversely, mediastinal germ cell tumor (MGCT) represents a small percentage of GCTs arising from the anterior mediastinum [4]. They represent <5% of all GCTs and <4% of all mediastinal neoplasms [5]. Nonseminomatous MGCT represents a more aggressive variant with a five-year overall survival rate of nearly 50%; therefore, they have been placed in the “poor risk” category by the updated analysis of the International Germ Cell Cancer Collaborative Group (IGCCCG) [6].

Up to seven different classes of GCTs can be identified according to their developmental potential [7]. This classification allows us to distinguish the categories of affected patients and clinical manifestation of the disease. In particular, GCTs affecting neonates and young children can be both benign or malignant (i.e., yolk sac tumors) manifestations, while tumors affecting adolescent and young adults (mean age 35 years) are mainly malignant. In this review we will focus on GCTs in adolescents and young adults.

Histologically, GCTs can be classified in two variants: seminomatous (S) and nonseminomatous (NS). While seminomas are composed of homogeneous cells, NS can be composed of several different histologies, with various degrees of differentiation: embryonal carcinoma, yolk sac tumor, choriocarcinoma and teratoma.

All these histologies can be found in GCTs; however, some types are more frequently detected in specific contexts or tumor localization. For example, seminoma is the most common type of TGCT.

Moreover, common biochemical features (alfa-fetoprotein, beta hCG, LDH) and molecular alterations (such as the gain of chromosome 12p) can be identified in both TGCTs and MGCTs. However, unlike NS-TGCTs, in which a high cure rate is reached upon platinum-based chemotherapy in most cases, NS-MGCTs are predominantly platinum-resistant and have a poor prognosis [6,8,9].

The young age of GCT patients makes the need to find effective therapeutic alternatives even more urgent for platinum-resistant subjects.

In this review, we aim to deepen our understanding of MGCTs, elaborating on their molecular similarities and disparities with TGCTs, as well as on the implications for the clinical management of these patients.

## 2. Origins

The genetic and epigenetic profile of GCTs is strictly connected to the cell of origin of these tumors. It is now widely accepted that malignant GCTs are not initiated by somatic mutations, but mostly by an alteration of the developmental fate of their cell of origin. Germ cell neoplasia in situ (GCNIS), found in the adjacent parenchyma of most GCTs, is the noninvasive precursor of germ cell tumors [10]. These cells have features resembling primordial germ cells (PGCs) or gonocytes, including expression of pluripotency markers such as OCT3/4 and NANOG, low DNA methylation level. During embryogenesis, PGCs originate from embryonic stem cells and are required for the germ line specification process. A delayed maturation of PGCs or of gonocytes can lead to GCNIS formation and then to initiation of the GCT [7,11,12].

**Figure 1:**
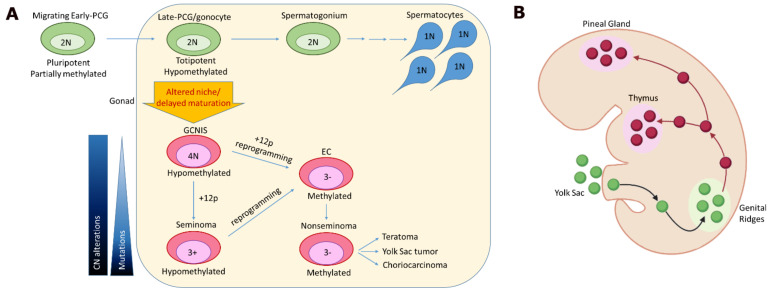
Origin of GCTs. (**A**) Schematic representation of TGCT development starting from early migrating PGCs during embryonal growth. PGCs start their epigenetic reprogramming and migrate from the yolk sac to the genital ridges. Once they have reached their niche, PGCs finish their epigenetic reprogramming through a global hypomethylation, and become gonocytes. Normally, gonocyte are diploid (2N) and differentiate in spermatogonium, which will give origins to spermatocytes (1N) after puberty. In the pathological setting, late-PGCs or gonocytes can double their genome (4N) and initiate a precursor lesion, defined as GCNIS. GCNIS, through the accumulation of additional copy number (CN) alterations (including gain of chromosome 12p), will then give origin to seminoma tumors after puberty. GCNIS and seminoma cells could also de-differentiate into ECs, through epigenetic reprogramming. ECs are characterized by a methylated genome, similar to embryonal stem cells, and have the pluripotency to give rise to the nonseminomatous histologies. Somatic mutations are scarce, acquired in the later steps of tumor progression. (**B**) Schematic representation of the migratory process of PGCs during embryonal development. Green and red dots represent respectively PGCs correctly migrating to the genital ridge and PGCs mismigrating and proliferating into the surrogate niches of the thymus or of the midline of the brain.

In the normal development process, during embryonic growth, early PGCs migrate from the yolk sac to their physiological niche (genital ridge) (future gonads) where they can finish their differentiation process to gonocytes (Figure 1A). Only after the birth do gonocytes differentiate to the spermatogonium state, which will terminally differentiate to spermatocytes after puberty.

In the pathological setting, PGC/gonocytes blocked in their maturation are able to originate the precursor lesion GCNIS, which remain quiescent until puberty. GCNIS has a double genome and retains the hypomethylated pattern of PGC. Polyploidy and demethylation state contribute to chromosome instability, and several losses and gains are frequent in GCNIS [13]. It is estimated that all GCNIS lesions will eventually transform into malignant GCTs [14].

Seminomatous tumors have histologic, methylation and expression features resembling PGC and GCNIS, and are considered to originate from these cells. Conversely, nonseminomatous tumors derive from embryonal carcinoma (EC) which originate from the transformation (through epigenetic reprogramming) of GCNIS or of seminoma cells. In turn, ECs, having the potential of an embryonal stem cell, are able to differentiate into the several lineages characteristic of nonseminomatous GCT (yolk sac, choriocarcinoma, teratoma and in rare cases heterologous differentiation) [15,16].

One of the key factors needed for GCT transformation is presumed to be located in the amplified chr12p. In fact, gain of the short arm of chr12 is considered a universal characteristic of all malignant GCTs, while it is generally not present in the precursor lesion GCNIS [10,12].

A better comprehension of TCGT development is provided by a study of the molecular heterogeneity and clone selection conducted on 4 primary nonseminomatous TGCTs, GCNIS and metastasis. These results allowed for the definition of a model of TGCTs evolution: GCNIS early doubled its genome and after puberty some GCNIS clones acquired genomic imbalances, including 12p gain. The clones carrying 12p are presumed to be the precursor with the invasive potential. Additional chromosomal loss and gain will contribute to the determination of the various histologies. Chromosome imbalances are frequent in GCNIS and GCTs, whereas somatic mutations are scarce and probably limited to the late stage of tumour development [13]. In fact, another evolutionary-based analysis conducted on a wider cohort demonstrated copy number alterations (CNAs) which arose earlier with respect to mutation in both primitive and metastatic samples [17].

Concerning extragonadal GCTs, it is widely accepted that they originate from an erroneous proliferation of mismigrated PGCs, the same precursor of gonadal tumors (Figure 1B) [18]. During the migration process to the genital ridge, PGCs could fail to exit from the nerve branches and continue migration through the midline of the body. These mismigrating cells could reach other surrogate niches located in the thymus or in the midline of the brain. Misplaced cells generally are eliminated by apoptosis; however, in the pathological setting, apoptosis is inhibited and the growth and transformation of PGCs is allowed. Eventually, these cells will give origin to extragonadal seminoma or nonseminoma GCTs [7].

Risk factors involved in the onset of GCT are still to be properly defined, even if a concerted role of both genetic and environmental factors is thought to be involved. A relevant role in TGCTs genesis is played by genetic factors and an association with disorders in sex developments (DSD) has been reported. Concerning MGCTs, Klinefelter’s syndrome is considered a risk factor [19]. However, a highly penetrant susceptibility gene for GCTs has not been identified. Conversely, it is thought that multiple, common, low-penetrance alleles form the basis for GCT familial syndrome [20]. Until now, 44 risk loci for TGCT onset have been identified, including SNPs involving KIT-KITL pathway, DNA damage repair (e.g., BRCA1), apoptosis (e.g., CHEK2), sex determination, telomeres and centrosome organization (e.g., TERT and CENPE) [21,22,23,24]. In addition to genetics, testicular dysgenesis syndrome is an important environmental factor associated with increased risk of TCGTs. In particular, this syndrome includes cryptorchidism, hypospadias, testicular atrophy, inguinal hernia and impaired spermatogenesis. More generally, a hypo-virilized gonad of the male embryo in utero may be involved in the genesis of GCTs [7,25].

## 3. Comparison between MGCTs and TGCTs Molecular Alterations

As stated above, the family of GCTs includes several different histologies, and this heterogeneous composition makes the molecular determinations quite difficult. Despite this, with the development of next generation sequencing (NGS) technologies, several integrated multi-omics studies have been conducted, which have made it possible to deepen our knowledge of the most frequent alterations in these tumors (Table 1).

A well-known feature of malignant GCTs, both mediastinal and testicular, and both seminoma and non-seminoma, is a gain of the short arm of chromosome 12, which frequently leads to the formation of an isochromosome (i12p). Interestingly, 12p gain is present in the great majority of post-puberal malignant GCTs (87% of cases) [16], while it is not detectable in GCTs affecting young children [26,27,28,29,30]. Moreover, even if some alterations have been reported in the precursor lesion, the gain of chromosome 12p tends to be acquired only later during TGCT development [12,13]. Many works have tried to identify the gene located in the 12p responsible for the pathogenesis of GCTs; however, a unique putative driver gene has not been identified. Of note, this region contains *KRAS* and *CCND2* (genes found mutated and/or overexpressed in TGCTs) and *NANOG and STELLAR* (genes involved in stem-cell lineage) [31,32]. Even if *KRAS* can be found mutated in TGCTs with chromosome 12p gain, the two events seem to be independent. One patient was reported carrying 12p gain on both GCNIS and in primary TGCT, while *KRAS* mutation was detected only in the TGCT, suggesting the two alterations are separate processes acquired during tumor evolution [33].

Globally, all GCTs show a low mutation rate, with approximately 0.5 mutations per Mb. Conversely, CNAs are frequent. TGCTs are generally hypertriploid, with NS-GCTs showing a slightly lower ploidy than seminomas. This state is a consequence of the early whole genome duplication, followed by the deletion of specific chromosome arms. Typically, a gain of 7, 8, 12, 21 and X chromosomes, and a loss of 1p, 11, 13 and Y are frequent in all TGCTs. Chromosome 12p gain tends to be more frequent in nonseminomas. NS-GCT have fewer copies of chromosomes 8, 9, 15, 19, 22, while seminomas have fewer copies of 11q. Moreover, focal amplification of *KRAS*, *KIT* and *MDM2* are frequent in TGCTs, regardless of the histology [16].

At the mutational level, S-TGCTs are enriched in mutations affecting *KIT* (18–25% of cases), *KRAS* (14%) or *NRAS* (4%) [16,34]. Moreover, deletion of *CBL* (a negative regulator of KIT) can be recurrently found in KIT wild type seminomas [16]. On the other side, NS-TGCTs show a different mutational profile and no significantly enriched somatic mutations identified [16,35,36]. *KIT* mutations are extremely rare in NS (2% of cases) [34], while RAS mutations are present in a minority of cases with respect to seminoma [16,34,35,37]. Other mutations have been reported in sporadic cases of NS-TGCT affecting *TP53*, *PIK3CA*, *AKT1* and *FGFR3* and they have been associated with cisplatin resistance [37,38].

Regarding epigenetics, seminoma and NS-GCTs have divergent methylation profiles, which reflect the different methylation states of the two histologies’ cells of origin. CpGs islands tend to be fully demethylated in seminoma, predominantly in KIT/RAS mutated cases. In KIT/RAS wild type seminoma, a residual methylation could be detected [16]. PGCs are known to be demethylated (a step necessary for the normal gametogenesis), and this similarity further supports the hypothesis of PGC/GCNIS as the cells of origin for seminoma [11,16]. Conversely, NS-GCTs are partially methylated: nonseminomas with a predominant EC composition tend to have a CpG profile similar to embryonic stem cell, while other NS-GCT (teratoma, yolk sac or mixed) have a stroma-like methylation profile [16]. This supports the origins of NS-GCTs, in which an un-methylated GCNIS or seminoma is reprogrammed to EC, which can re-establish the level of methylation and possibly differentiates into the various NS components.

MGCTs are mainly diploid, but have several common molecular alterations with TGCTs supporting a common origin of the two types of tumors. A recent pan cancer study identified that, similarly to S-TGCTs, mutations in *KRAS*, *NRAS* and *KIT* were significantly associated with extragonadal GCTs (mediastinal and retroperitoneal) [34]. *KIT* mutations predominantly affected exon 17 and led to the activation of the protein [34,39]. Moreover, an NGS study conducted on 44 primary MGCTs showed that these tumors, similarly to TGCTs, have a low mutational burden and confirm the presence of alterations on the RAS pathway (*KRAS*, *NRAS*). In this cohort, composed of only nonseminomatous tumors, *KIT* alterations were almost absent. This data is consistent with the lower frequency of *KIT* mutations in all NS-GCTs [40]. On the other hand, differing from TGCTs, MGCTs showed an increase in rate of *TP53* and *PTEN* mutations [34,40].

The high frequency of *TP53* alterations in MGCTs in comparison with TGCTs is noteworthy. Mutations of *TP53* were reported in approximately 82% of NS-MGCT cases with respect to 4% of S-TGCTs and 20% of NS-TGCTs [40]. The presence of *TP53* pathway alterations in almost all cases of MGCT is presumed to be one of the reasons behind the poorer prognosis of this type of tumor. On the contrary, TGCTs generally retain the activity of *TP53*. Wild type *TP53* is considered one of the factors behind the high sensitivity of TGCT to platinum-based therapies, which in concertation with other factors (i.e., high mitochondrial priming and defective homologous recombination for induction of apoptosis of irreparably damaged cells) is involved in the control and maintenance of genetic stability [16,33]. The ability of TGCT to induce apoptosis in damaged cells reflects the intrinsic characteristic of the cell of origin of TCGT, the PGC, in which these mechanisms of control are necessary for limiting the risk of new mutations being passed on to future generations [7]. In support of this hypothesis, *TP53* mutations are almost absent in TGCTs, and the protein is highly expressed [16], while an increased mutation rate of *TP53* has been reported in NS-TGCT resistant to platinum-based therapies [37].

## 4. MGCT: Clinico-Pathological Features and Treatment

As previously said, the mediastinum represents a site of poor prognosis, and primary GCTs arising from it are commonly resistant to standard and high-dose chemotherapy regimens. Usually, they are found incidentally on a chest radiological evaluation due to their asymptomaticity, especially at the first period. Then, when their size increases, they become symptomatic due their compressive/obstructive effect on nearby organs or vessels. The most common symptoms are actually dyspnea, hyperthermia, cough, chest pain, weight loss and superior vena cava syndrome [41].

Different GCT histologies can be found in the mediastinum. Usually, seminoma represents a portion of the tumor, in case of mixed histology, but those tumors must be considered and treated as non-seminomas [8]. An elevation of beta-hCG serum levels is found in a 30% of cases, whereas an elevation of alpha- fetoprotein excludes the presence of a pure seminoma [42].

Yolk sac tumor histology represents approximately 60% of all non-seminomatous MGCT cases: these tumors are constituted by solid masses with necrotic and hemorrhagic areas [43]. The presence of wide hemorrhagic zones is also a characteristic of choriocarcinomas, which are usually larger tumors, with different early blast cells that are typical of the embryogenesis period [43,44,45].

The rarity of these tumors has meant that to date there are no large prospective studies able to establish specific treatment modalities; therefore, the International Guidelines recommend using the same therapy schemes validated for tumors of gonadal origin. In particular, according to the IGCCCG Guidelines [6], NS-MGCTs are treated as “poor risk” neoplasms, whereas seminoma is considered as good or intermediate risk based on LDH expression levels and presence of metastases [46]. A meta-analysis conducted in a cohort of 1800 NS-GCT patients who underwent first-line chemotherapy from 1989 to 2002 demonstrated a similar five-year survival rate for the good and intermediate prognostic group (71%) instead of 48% reported in the poor-risk group [47]. In recent years, the spread of high-dose treatment [48,49,50] after the first line has led to an improvement in terms of outcome also in the **“**poor risk**”** category, so much so that a new scoring system has recently been introduced for these patients [51]. This new classification gives a poor prognosis to visceral metastases, even though primary MGCTs represent the worst prognosis cases, regardless of the treatment they undergo [51].

NS-MGCT patients usually undergo chemotherapy, followed by surgery of residual tissue, when feasible. The number of chemotherapy courses depends on the specific IGCCCG category risk. Usually, the most frequently used regimen is a combination of bleomycin, etoposide, and cisplatin (BEP), in which bleomycin is substituted by ifosfamide with an aim to avoid its lung toxicity. Notwithstanding, better survival rates were reported in a 28-patient cohort treated with high-dose ifosfamide instead of bleomycin [52].

With regards to benign mature teratomas, which do not respond to chemotherapy, they have to be treated with surgery. These patients undergo a median sternotomy or posterolateral thoracotomy, and even in the case of non-radical surgery, they do not require postoperative chemotherapy, given their low propensity for local invasiveness or metastatisation.

After chemotherapy, growing teratoma syndrome is a rare effect reported in unresected tissue: the malignant mass is comprised only of mature teratomatous cells, probably selected by the chemotherapeutic regimen [4]. A new resection is recommended in selected patients, given that it may improve long-term survival [53].

Concerning tumors classified as poor prognosis, most of them will relapse. A study involving 79 patients reported a long-term disease-free survival rate of 11%. Therefore, for many of these patients a second line of chemotherapy is envisaged, with respect to which, in the international guidelines, no specific regimen seems to prevail over the others, both at standard and high doses [54,55,56,57,58,59]. In order to optimize care for these patients, several potential prognostic and predictive factors have been analyzed in recent years, including systemic inflammatory index (SII) [60,61,62], neutrophil-to-lymphocyte ratio (NLR) [63,64], platelet-to-lymphocyte ratio (PLR) [60]. However, whatever the choice, the response rate is currently very low, and is between 5 and 10% [42].

## 5. MGCT and Concomitant Neoplasms

One negative but peculiar aspect of primary MGCT is their association with the concomitant occurrence of other somatic malignancies, including sarcoma, carcinoma and several types of hematologic malignancies (HMs). These last ones are the most common concomitant neoplasms detected in MGCT patients [42,65].

In a multicenter study analysis 635 extragonadal GCTs reported the development of HMs in about 2–3% of cases: all GCTs associated with HMs were localized in the mediastinum and are of the nonseminomatous type. HMs have been reported to arise concomitantly or within a median of 6 months from primary MGCT. Considering only MGCTs, the incidence of concomitant HMs is about 6% and this condition is marked by an extremely poor outcome [65].

The first evidence of the association between MGCT and synchronous HM was reported in 1985 [66] and until now several case reports and few case series have been documented [67]. Even if few cases with seminomatous components were reported, the almost totality of MGCTs associated with secondary HMs are generally NS, with yolk sac or mature teratoma components. On the other hand, the most frequent type of HM is acute myeloid leukemia (AML), predominantly with megakaryoblastic differentiation (M7). Other reported secondary HM includes myelodysplastic syndromes, chronic myeloid leukemia, malignant histiocytosis and essential thrombocytopenia [67,68,69].

Initially the hematologic manifestation was presumed to be a secondary complication induced by the chemotherapy. However, the presence of synchronous manifestations (before chemotherapy) and the increased number of molecular investigations performed on paired primary MGCT e hematologic tumors suggest the presence of a common origin for the two diseases (Table 2) [68,69,70].

Evidence of a clonal relationship between HM and MGCT was discovered through the identification of molecular alterations shared between the two tumor types (i12p/12p gain, *TP53*, *KRAS* and *PTEN* mutations) (Table 1) [66,68,69,70,71,72,73,74,75,76,77,78,79,80]. Moreover, the hematologic disease frequently carries alterations not typical of the specific type of HM and lacks the canonical aberrations detected in de novo diseases. For example, gain or isochromosome of 12p is an event not detectable in canonical AML [69], but it has been reported in 18 out of 38 HMs associated with MGCTs (approximately 47% of cases) (Table 2) [66,68,69,70,71,72,73,74,76]. Conversely common alterations, including MLL rearrangements or mutations on genes such as *FLT3*, *NPM1* and others, were not detected in AML coupled with MGCTs [67,69,72,77,78].

However, whether the HM derives from a differentiation of the totipotent GCT tumor mass or from a shared precursor is still debated. Orazi et al. hypothesised that the yolk sac and the teratoma component of the GCT could have the ability to migrate to the bone marrow and to differentiate in the hematologic lineage [68]. However, a recent effort by Taylor and colleagues [69] tried to clarify this point, performing comprehensive genomic analysis of a wide case series of MGCTs presenting one, or more, concomitant HM. In particular, mutation and CNA analysis of MGCTs and concomitant HMs in 5 patients allowed them to infer the evolutionary relationship between the two malignancies and to identify the genetic profile of the common precursor. In most cases, *TP53* or RAS alterations were detected in the common precursor, and subsequent private acquired alterations were reported in each different disease (MGCT or HM). This demonstrates that a common founder clone, probably a PGC located in the mediastinum and reprogrammed to EC, is responsible for the parallel development of MGCT and HM. This precursor has common genetic alterations retained by the two types of tumors (i.e., *TP53* and RAS), then, a subset of clonally acquired alterations can characterize each specific disease evolution [69].

Considering the entire cohort analyzed by Taylor et al., the most frequently altered gene was *TP53*, which affects 91% of MGCTs with secondary HMs [69]. This is consistent with the previous case reports of NGS analysis in which *TP53* alterations were detected in all cases tested (Table 1) [72,73,74,75,76,77]. Interestingly, the frequency of *TP53* is slightly greater than the one reported in all NS-MGCTs regardless of the HM onset (82% cases) [40]. As stated above, *TP53* alterations contribute to the worse prognosis of these patients, since they confer resistance to both GCT- and HM-directed therapies.

In addition, RAS alterations (*KRAS*/*NRAS*) also seem to be more frequent in MGCTs associated with HMs (63% vs. 37–45% reported in canonical MGCTs) [40,69]. Conversely, the frequency of *PTEN* mutations, which were frequently reported in cases reports [73,74,75], is similar between MGCTs with or without HM [40,69]. Of note, mutational frequencies in MGCTs with secondary HMs are only an estimation, since they are based on the cohort studied by Taylor et al. that include NGS analysis of only 12 patients. This is the largest cohort analyzed so far, but the rarity of this complex disease hinders the study of a larger case series.

Globally, even if the genotype of the precursor clone has been studied, the alterations detected in the MGCT could only partially explain the onset of the secondary HM. The high frequency of *TP53* and RAS mutations in MGCTs with concomitant HMs, and the presence of these alterations in the common precursor clone, leads researcher to suppose that *TP53*/RAS pathway alterations are necessary for PGCs to survive in the extragonadal niche [69,79]. However, whether other factors can be implied to this peculiar disease is still to be investigated. The only recognized risk factor is the presence of Klinefelter syndrome, which is associated with a higher incidence of MGCTs [19,80]. Interestingly, one recent study indicates that the presence of post-chemotherapy vasculogenic lesions in the tumor could be a risk factor for the onset of leukemia or myelodysplasia in MGCTs [81]. Despite all these advances at the molecular and pathological levels, further studies will be needed to assess the biological factors involved in the development of concomitant hematological disorders.

## 6. Final Remarks and Future Perspectives

Mediastinal GCT is a rare disease with an extremely poor prognosis. Even if the origin of MGCTs and their mutational profile have similarities to TGCTs, the differences in disease presentation make the mediastinal tumors more difficult to treat by canonical GCT chemotherapeutic regimens. Moreover, the concomitant onset of hematologic disease, occurring in a small percentage of MGCTs, makes the disease even more aggressive.

Despite recent advances in GCT molecular characterization, several unsolved issues remain.

Currently, very limited treatment options are available for refractory GCTs. Clinical trials on target therapies produced poor results [82], while the low TMB and the low percentage of T-cell infiltrates in NS tumors seem to hamper the use of immunotherapy [83,84,85].

Moreover, there is still the need to identify biomarkers predictive of tumor resistance to chemotherapies. The high incidence of *TP53* mutations reported in MGCTs is indicated as one probable factor responsible for the poor sensitivity of MGCT to therapies, however no alternative therapeutic strategies are currently available. It may be the case that integrated studies including epigenetic and transcriptional analysis will enhance our comprehension of these tumors and highlight novel therapeutic targets. In addition, the study of free circulating cells or molecules in plasma could constitute a novel method for biomarker discovery. In particular, it has been shown that detection of free circulating tumor cells correlates with tumor stage and aggressiveness in several tumors, including GCT [86,87,88]. Further studies will be needed to assess the predictive value of this approach.

Finally, even if secondary HMs have been demonstrated to originate from a precursor shared with the primary tumor, the reason behind their onset only in the context of mediastinal tumors is still unknown. The implementation of proper disease models is necessary for the understanding of these mechanisms.

The rarity and complexity of the mediastinal disease hamper the research process of finding novel therapeutic approaches. Until then, referral centers with experience in mediastinal tumors should be involved in the management of these patients.

## Figures and Tables

**Table 1:** Frequency of the most recurrent mutations identified in TGCT and MGCT.

Gene	GCT		TGCT		MGCT
	Sensitive	Resistant	Seminomas	Nonseminomas	All
*KIT*	17%	4%	18–25%	2%	na
*RAS*	14–18%	12%	18%	9%	37%
*TP53*	0%	16%	4%	20%	61–82%

na: not available.

**Table 2:** Molecular analysis of MGCTs with concomitant secondary HM. All English-language literature published until August 2021 containing molecular analysis on MGCT patients developing concomitant HM were searched in PubMed. In the table are reported only the patients for which molecular testing was performed in at least one sample (primary MGCT or HM). Cytogenetics, direct sequencing, array or NGS analysis were considered. The most relevant/recurrent alterations reported are shown.

Refs.	Histology ^1^		Molecular Analysis ^2^		Molecular Alterations ^3^	
	MGCT	HM	MGCT	HM	MGCT	HM
[66]	na	AML M7	na	CG	na	-
	na	AML M7	na	CG	na	-
	na	AML M7	na	CG	na	-
	na	AML M7	na	CG	na	-
	na	AML M5	na	CG	na	-
	na	AUL	na	CG	na	i12p
	na	ET	na	CG	na	-
	na	ET	na	CG	na	-
	na	MM	na	CG	na	-
	na	MM	na	CG	na	-
[68]	MT, Y	AML M6 + M7	na	CG	na	-
	MT, Y	AML M4	na	CG	na	i12p
	MT, Y	AML M6	na	CG	na	i12p
	MT, Y, SARC	AML M7	na	CG	na	-
	MT, IT, Y	AML M7	na	CG	na	-
[70]	Y, IT, SARC	AML M2	CG	CG	i12p	i12p
[71]	Y, IT	AML M4	na	CG	na	-
	IT, S, Y	AML M4	na	CG	na	-
	IT	AML M5	na	CG	na	i12p
[72]	MT+YS	MDS; AML M2+M6	na	CG	na	i12p
[73]	mixed	AML M1	na	CG	na	i12p
	MT, IT	AML M0	na	CG	na	i12p
	mixed	MS	CG	CG	i12p	i12p
[74]	na	AML M7	WES	WES	**+12p,** ***TP53, PTEN***	**+12p,** ***TP53, PTEN***
[75]	MT+SARC	AML M6	sanger	CG+TS	***TP53, NRAS***	***TP53, NRAS***
[76]	mixed	AML M7	array + TS	CG + array +TS	**i12p,** ***TP53, PTEN***	**i12p,** ***TP53, PTEN***
[77]	IT, Y	AML M7	CG+WES	CG+WES	**+12p,** ***TP53, PTEN***	**+12p,** ***TP53, PTEN***
[78]	MT, SARC	AML M7	CG+WES	CG+WES	**+12p,** ***KRAS*,** ***TP53, PTEN***	**+12p,** ***KRAS*,** ***TP53, PTEN***
[69]	T	Mastocytosis	TS	CG	TP53, PTEN	i12p
	Y, T	MDS + HS	WES	WES +CG (all)	**i12p,** ***RRAS2*,** ***BCOR*,** ***TP53***	**MDS: i12p,** ***RRAS2*,** ***BCOR*,** ***TP53*; HS:** ***RRAS2*,** ***BCOR*,** ***TP53* ^4^,** ***NRAS*.**
	T, Y	HS + CMML + AML M7	WES + CG	WES + CG (all)	***TP53*,*PIK3CD***, +1q, +21q	**AML:*TP53*,*PIK3CD***, +1q, +21q, −7q; HS and CMML: ***TP53*,*PIK3CD*,*NRAS***
	T, Y	AML (non-M7) + MDS	na	TS	na	*TP53*
	T	AML (non-M7)	TS	na	***TP53*,** ***NRAS*,** ***RRAS2***	na
	Y, T	MDS + AML M7	WES + CG	WES +CG (MDS)	**i12p,** ***KRAS*,** ***TP53* ^4^,** ***ARID1A***	**i12p,** ***KRAS*,** ***TP53* ^4^**
	CC	MDS	WES	WES+CG	***TP53***	***TP53***
	T	HS	na	TS	na	***TP53*,** ***KRAS***
	na	HLH	na	TS	na	***TP53*,** ***PTEN*,** ***NRAS***
	S, E	TCL + HLH	TS	TS (TCL)	***TP53*,** ***PTEN***	***TP53***
	T, Y	CMML+ HLH	WES	WES +CG (CMML)	**i12p,** ***KRAS*,** ***AKT1***	**i12p,** ***KRAS*,** ***MED12***
	Y	AML M7	CG	CG	**i12p**	**i12p**

^1^ MGCT histologies: CC = Choriocarcinoma; T = Teratoma; MT = Mature Teratoma; IT = immature teratoma, S = Seminoma, Y = Yolk sac tumor, E = Embryional, SARC = sarcomatous components, unk = unknown; HM histologies: TCL = T-cell lymphoma, MDS = Myelodysplastic syndrome, HS = Histiocytic sarcoma, CMML = Chronic myelomonocytic leukemia, HLH = Hemophagocytic Lymphohistiocytosis, AML = Acute myeloid leukemia, MS = Myeloid sarcoma, ET = essential thrombocythemia, MM = magakariocystic myelosis, AUL = Acute undifferentiated leukemia; na = data not available or analysis not performed; ^2^ WES = Whole exome sequencing, TS = target or panel gene sequencing, GC = Cytogenetics analysis (Karyotype or FISH); na = data not available or analysis not performed; ^3^ Alterations shared between GCT and HM highlighted in bold; dashed line (-) indicate no relevant/recurrent alterations reported, while “na” indicates data not available or analysis not performed; ^4^ TP53 mutation type (aminoacidic position) differs between MGCT and HM. Alterations shared between GCT and HM highlighted in bold.

## References

[1] Rusner C., Trabert B., Katalinic A., Kieschke J., Emrich K., Stang A. (2013). Network of German Cancer Registries (GEKID). In-cidence patterns and trends of malignant gonadal and extragonadal germ cell tumors in Germany, 1998–2008. Cancer Epidemiol..

[2] Scholz M., Zehender M., Thalmann G.N. (2002). Extragonadal retroperitoneal germ cell tumor: Evidence of origin in the testis. Ann. Oncol..

[3] Fizazi K., Oldenburg J., Dunant A., Chen I., Salvioni R., Hartmann J.T., De Santis M., Daugaard G., Flechon A., de Giorgi U. (2007). Assessing prognosis and optimizing treatment in patients with postchemotherapy viable nonseminomatous germ-cell tumors (NSGCT): Results of the sCR2 international study. Ann. Oncol..

[4] Kang J., Mashaal H., Anjum F. (2021). Mediastinal Germ Cell Tumors. StatPearls [Internet].

[5] Mishra S., Das Majumdar S.K., Sable M., Parida D.K. (2020). Primary malignant mediastinal germ cell tumors: A single institutional experience. S. Asian J. Cancer.

[6] Gillessen S., Sauvé N., Collette L., Daugaard G., de Wit R., Albany C., Tryakin A., Fizazi K., Stahl O., Gietema J.A. (2021). Predicting Outcomes in Men With Metastatic Nonseminomatous Germ Cell Tumors (NSGCT): Results from the IGCCCG Update Consortium. J. Clin. Oncol..

[7] Oosterhuis J.W., Looijenga L.H.J. (2019). Human germ cell tumours from a developmental perspective. Nat. Rev. Cancer.

[8] Aparicio J., Terrasa J., Durán I., Germà-Lluch J.R., Gironés R., González-Billalabeitia E., Gumà J., Maroto P., Pinto A., García-Del-Muro X. (2016). SEOM clinical guidelines for the management of germ cell testicular cancer (2016). Clin. Transl. Oncol..

[9] Fizazi K., Culine S., Droz J.P., Kramar A., Théodore C., Ruffié P., Le Chevalier T. (1998). Primary mediastinal nonseminomatous germ cell tumors: Results of modern therapy including cisplatin-based chemotherapy. J. Clin. Oncol..

[10] Skakkebæk N.E., Berthelsen J.G., Giwercman A., Müller J. (1987). Carcinoma-in-situ of the testis: Possible origin from gonocytes and precursor of all types of germ cell tumours except spermatocytoma. Int. J. Androl..

[11] Rijlaarsdam M., Tax D.M.J., Gillis A.J.M., Dorssers L.C.J., Koestler D..C., de Ridder J., Looijenga L.H.J. (2015). Genome Wide DNA Methylation Profiles Provide Clues to the Origin and Pathogenesis of Germ Cell Tumors. PLoS ONE.

[12] Looijenga L.H., de Munnik H., Oosterhuis J.W. (1999). A molecular model for the development of germ cell cancer. Int. J. Cancer.

[13] Dorssers L.C.J., Gillis A.J.M., Stoop H., van Marion R., Nieboer M.M., van Riet J., van de Werken H.J.G., Oosterhuis J.W., de Ridder J., Looijenga L.H.J. (2019). Molecular heterogeneity and early metastatic clone selection in testicular germ cell cancer development. Br. J. Cancer.

[14] Kier M.G.G., Lauritsen J., Almstrup K., Mortensen M.S., Toft B.G., Rajpert-De Meyts E., Skakkebaek N.E., Rørth M., von der Maase H., Agerbaek M. (2015). Screening for carcinoma in situ in the contralateral testicle in patients with testicular cancer: A population-based study. Ann. Oncol..

[15] Honecker F., Stoop H., Mayer F., Bokemeyer C., Castrillon D.H., Lau Y.-F.C., Looijenga L., Oosterhuis J.W. (2005). Germ cell lineage differentiation in non-seminomatous germ cell tumours. J. Pathol..

[16] Shen H., Shih J., Hollern D.P., Wang L., Bowlby R., Tickoo S.K., Thorsson V., Mungall A., Newton Y., Hegde A.M. (2018). Integrated Molecular Characterization of Testicular Germ Cell Tumors. Cell Rep..

[17] Cheng M.L., Donoghue M.T., Audenet F., Wong N.C., Pietzak E.J., Bielski C.M., Isharwal S., Iyer G., Funt S., Bagrodia A. (2020). Germ Cell Tumor Molecular Heterogeneity Revealed Through Analysis of Primary and Metastasis Pairs. JCO Precis. Oncol..

[18] Chaganti R., Rodriguez E., Mathew S. (1994). Origin of adult male mediastinal germ-cell tumours. Lancet.

[19] Hasle H., Mellemgaard A., Nielsen J., Hansen J. (1995). Cancer incidence in men with Klinefelter syndrome. Br. J. Cancer.

[20] Greene M.H., Mai P.L., Loud J.T., Pathak A., Peters J.A., Mirabello L., McMaster M.L., Rosenberg P., Stewart D.R. (2014). Familial testicular germ cell tumors (FTGCT)—Overview of a multidisciplinary etiologic study. Andrology.

[21] Looijenga L.H., Hersmus R., Oosterhuis J.W., Cools M., Drop S.L., Wolffenbuttel K.P. (2007). Tumor risk in disorders of sex development (DSD). Best Pract. Res. Clin. Endocrinol. Metab..

[22] Litchfield K., Levy M., Orlando G., Loveday C., Law P.J., Migliorini G., Holroyd A., Broderick P., Karlsson R., Haugen T.B. (2017). Identification of 19 new risk loci and potential regulatory mechanisms influencing susceptibility to testicular germ cell tumor. Nat. Genet..

[23] Wang Z., A McGlynn K., Meyts E.R.-D., Bishop D.T., Chung C.C., Dalgaard M.D., Greene M.H., Gupta R., Grotmol T., Haugen T.B. (2017). Meta-analysis of five genome-wide association studies identifies multiple new loci associated with testicular germ cell tumor. Nat. Genet..

[24] Aldubayan S.H., Pyle L.C., Gamulin M., Kulis T., Moore N.D., Taylor-Weiner A., Hamid A.A., Reardon B., Wubbenhorst B., Godse R. (2019). Association of Inherited Pathogenic Variants in Checkpoint Kinase 2 (CHEK2) With Susceptibility to Testicular Germ Cell Tumors. JAMA Oncol..

[25] Skakkebaek N.E., Rajpert-De Meyts E., Main K.M. (2001). Testicular dysgenesis syndrome: An increasingly common developmental disorder with environmental aspects. Hum. Reprod..

[26] Rodriguez E., Mathew S., Reuter V., Ilson D.H., Bosl G.J., Chaganti R.S. (1992). Cytogenetic analysis of 124 prospectively ascer-tained malegerm cell tumors. Cancer Res..

[27] Bussey K.J., Lawce H.J., Olson S.B., Arthur D.C., Kalousek D.K., Krailo M., Giller R., Heifetz S., Womer R., Magenis R.E. (1999). Chromo-some abnormalities of eighty-one pediatric germ cell tumors: Sex-, age-, site-, and histopathology-related differences—A children’s cancer group study. Genes Chromosomes Cancer.

[28] Kao C.-S., Bangs C.D., Aldrete G., Cherry A.M., Ulbright T.M. (2018). A Clinicopathologic and Molecular Analysis of 34 Mediastinal Germ Cell Tumors Suggesting Different Modes of Teratoma Development. Am. J. Surg. Pathol..

[29] Schneider D.T., Schuster A.E., Fritsch M.K., Hu J., Olson T., Lauer S., Göbel U., Perlman E.J. (2001). Multipoint imprinting analysis indicates a common precursor cell for gonadal and nongonadal pediatric germ cell tumors. Cancer Res..

[30] Schneider D.T., Schuster A.E., Fritsch M.K., Calaminus G., Göbel U., Harms D., Lauer S., Olson T., Perlman E. (2002). Genetic analysis of mediastinal nonseminomatous germ cell tumors in children and adolescents. Genes Chromosom. Cancer.

[31] Sperger J.M., Chen X., Draper J.S., Antosiewicz J.E., Chon C.H., Jones S.B., Brooks J.D., Andrews P.W., Brown P.O., Thomson J.A. (2003). Gene expression patterns in human embryonic stem cells and human pluripotent germ cell tumors. Proc. Natl. Acad. Sci. USA.

[32] Rodriguez S., Jafer O., Goker H., Summersgill B.M., Zafarana G., Gillis A.J.M., Van Gurp R.J.H.L.M., Oosterhuis J.W., Lu Y.-J., Huddart R. (2003). Expression profile of genes from 12p in testicular germ cell tumors of adolescents and adults associated with i(12p) and amplification at 12p11.2–p12.1. Oncogene.

[33] Taylor-Weiner A., Zack A.T.-W.T., O’Donnell E., Guerriero J.L., Bernard B., Reddy A., Han G.C., AlDubayan S.H., Amin-Mansour A., Schumacher S.E. (2016). Genomic evolution and chemoresistance in germ-cell tumours. Nature.

[34] Loveday C., Litchfield K., Proszek P.Z., Cornish A.J., Santo F., Levy M., MacIntyre G., Holryod A., Broderick P., Dudakia D. (2020). Genomic landscape of platinum resistant and sensitive testicular cancers. Nat. Commun..

[35] Litchfield K., Summersgill B., Yost S., Sultana R., LaBreche K., Dudakia D., Renwick A., Seal S., Al-Saadi R., Broderick P. (2015). Whole-exome sequencing reveals the mutational spectrum of testicular germ cell tumours. Nat. Commun..

[36] McIntyre A., Summersgill B., Spendlove H.E., Huddart R., Houlston R., Shipley J. (2005). Activating Mutations and/or Expression Levels of Tyrosine Kinase Receptors GRB7, RAS, and BRAF in Testicular Germ Cell Tumors. Neoplasia.

[37] Bagrodia A., Lee B., Lee W., Cha E.K., Sfakianos J.P., Iyer G., Pietzak E.J., Gao S.P., Zabor E.C., Ostrovnaya I. (2016). Genetic Determinants of Cisplatin Resistance in Patients With Advanced Germ Cell Tumors. J. Clin. Oncol..

[38] Feldman D.R., Iyer G., Van Alstine L., Patil S., Al-Ahmadie H., Reuter V.E., Bosl G., Chaganti R.S., Solit D.B. (2014). Presence of Somatic Mutations within PIK3CA, AKT, RAS, and FGFR3 but not BRAF in Cisplatin-Resistant Germ Cell Tumors. Clin. Cancer Res..

[39] Przygodzki R.M., Hubbs A.E., Zhao F.-Q., O’Leary T.J. (2002). Primary mediastinal seminomas: Evidence of single and multiple KIT mutations. Lab. Investig..

[40] Necchi A., Bratslavsky G., Chung J., Millis S., Gay L.M., Ali S.M., Ross J.S. (2018). Genomic Features for Therapeutic Insights of Chemotherapy-Resistant, Primary Mediastinal Nonseminomatous Germ Cell Tumors and Comparison with Gonadal Counterpart. Oncologist.

[41] Travis W.D., Brambilla E., Nicholson A.G., Yatabe Y., Austin J.H.M., Beasley M.B., Chirieac L.R., Dacic S., Duhig E., Flieder D.B. (2015). The 2015 world health organization classification of lung tumors: Impact of genetic, clinical and radiologic advances since the 2004 classification. J. Thorac. Oncol..

[42] Rosti G., Secondino S., Necchi A., Fornarini G., Pedrazzoli P. (2019). Primary mediastinal germ cell tumors. Semin. Oncol..

[43] De Giorgi U., Richard S., Badoglio M., Kanfer E., Bourrhis J.H., Nicolas-Virelizier E., Vettenranta K., Lioure B., Martin S., Dreger P. (2017). Salvage high-dose chemotherapy in female patients with relapsed/refractory germ-cell tumors: A retrospective analysis of the European Group for Blood and Marrow Transplantation (EBMT). Ann. Oncol..

[44] Domínguez Malagón H., Pérez Montiel D. (2005). Mediastinal germ cell tumors. Semin. Diagn. Pathol..

[45] Drevelegas A., Palladas P., Scordalaki A. (2001). Mediastinal germ cell tumors: A radiologic–pathologic review. Eur. Radiol..

[46] Beyer J., Collette L., Sauvé N., Daugaard G., Feldman D.R., Tandstad T., Tryakin A., Stahl O., Gonzalez-Billalabeitia E., De Giorgi U. (2021). International germ cell cancer classification update consortium. survival and new prognosticators in metastatic seminoma: Results from the IGCCCG-update consortium. J. Clin. Oncol..

[47] van Dijk M.R., Steyerberg E.W., Habbema J.D. (2006). Survival of non-seminomatous germ cell cancer patients according to the IGCC classification: An update based on meta-analysis. Eur. J. Cancer.

[48] De Giorgi U., Rosti G., Salvioni R., Papiani G., Ballardini M., Pizzocaro G., Marangolo M. (2011). Long-term outcome of salvage high-dose chemotherapy in patients with germ cell tumor with poor prognostic features. Urol. Oncol. Semin. Orig. Investig..

[49] Einhorn L.H., Williams S.D., Chamness A., Brames M.J., Perkins S.M., Abonour R. (2007). High-dose chemotherapy and stem-cell rescue for metastatic germ-cell tumors. N. Engl. J. Med..

[50] Kopf B., De Giorgi U., Vertogen B., Kopf B., De Giorgi U., Vertogen B., Monti G., Molinari A., Turci D., Dazzi C. (2006). A randomized study comparing filgrastim versus lenograstim versus molgramostim plus chemotherapy for peripheral blood progenitor cell mobilization. Bone Marrow Transplant..

[51] Lorch A., Beyer J., Bascoul-Mollevi C., Kramar A., Einhorn L.H., Necchi A., Massard C., De Giorgi U., Fléchon A., Prognostic Factors Study Group (2010). Prognostic factors in patients with metastatic germ cell tumors who experienced treatment failure with cisplatin-based first-line chemotherapy. J. Clin. Oncol..

[52] Bokemeyer C., Schleucher N., Metzner B., Thomas M.S.C., Rick O., Schmoll H.-J., Kollmannsberger C., Boehlke I., Kanz L., Hartmann J.T. (2003). First-line sequential high-dose VIP chemotherapy with autologous transplantation for patients with primary mediastinal nonseminomatous germ cell tumours: A prospective trial. Br. J. Cancer.

[53] Vuky J., Bains M., Bacik J., Higgins G., Bajorin D.F., Mazumdar M., Bosl G.J., Motzer R.J. (2001). Role of postchemotherapy adjunctive surgery in the management of patients with nonseminoma arising from the mediastinum. J. Clin. Oncol..

[54] Hartmann J.T., Einhorn L., Nichols C.R., Droz J.P., Horwich A., Gerl A., Fossa S.D., Beyer J., Pont J., Schmoll H.J. (2001). Second-line chemotherapy in patients with relapsed extragonadal nonseminomatous germ cell tumors: Results of an international multicenter analysis. J. Clin. Oncol..

[55] Voss M.H., Feldman D.R., Bosl G.J., Motzer R.J. (2011). A review of second-line chemotherapy and prognostic models for disseminated germ cell tumors. Hematol. Oncol. Clin. N. Am..

[56] Lorch A., Bascoul-Mollevi C., Kramar A. (2011). Conventional-dose versus high-dose chemotherapy as first salvage treatment in male patients with metastatic germ cell tumors: Evidence from a large international database. J. Clin. Oncol..

[57] De Giorgi U., Demirer T., Wandt H., Taverna C., Siegert W., Bornhauser M., Kozak T., Papiani G., Ballardini M., Rosti G. (2005). Second-line high-dose chemotherapy in patients with mediastinal and retroperitoneal primary non-seminomatous germ cell tumors: The EBMT experience. Ann. Oncol..

[58] De Giorgi U., Rosti G., Aieta M., Testore F., Burattini L., Fornarini G., Naglieri E., Re G.L., Zumaglini F., Marangolo M. (2006). Phase II Study of Oxaliplatin and Gemcitabine Salvage Chemotherapy in Patients with Cisplatin-Refractory Nonseminomatous Germ Cell Tumor. Eur. Urol..

[59] Adra N., Abonour R., Althouse S.K., Albany C., Hanna N.H., Einhorn L.H. (2017). High-dose chemotherapy and autologous peripheral-blood stem-cell transplantation for relapsed metastatic germ cell tumors: The indiana university experience. J. Clin. Oncol..

[60] Cursano M.C., Kopf B., Scarpi E., Menna C., Casadei C., Schepisi G., Lolli C., Altavilla A., Gallà V., Santini D. (2020). Prognostic Role of Systemic Inflammatory Indexes in Germ Cell Tumors Treated With High-Dose Chemotherapy. Front. Oncol..

[61] Chovanec M., Cierna Z., Miskovska V., Machalekova K., Kalavska K., Rejlekova K., Svetlovska D., Macak D., Spanik S., Kajo K. (2018). Systemic immune-inflammation index in germ-cell tumours. Br. J. Cancer.

[62] Fankhauser C.D., Sander S., Roth L., Gross O., Eberli D., Sulser T., Seifert B., Beyer J., Hermanns T. (2018). Systemic inflammatory markers have independent prognostic value in patients with metastatic testicular germ cell tumours undergoing first-line chemotherapy. Br. J. Cancer.

[63] Bolat D., Aydoğdu Ö., Polat S., Yarımoğlu S., Bozkurt İ.H., Yonguç T., Şen V. (2017). Predictive value of preoperative neutrophil-to-lymphocyte ratio on the prognosis of germ cell testicular tumors. Turk. J. Urol..

[64] Jankovich M., Jankovichova T., Ondrus D., Breza J. (2017). Neutrophil-to-lymphocyte ratio as a predictor of preoperative tumor staging in testicular germ cell tumors. Bratisl. Med. J..

[65] Hartmann J.T., Nichols C.R., Droz J., Horwich A., Gerl A., Fossa S.D., Beyer J., Pont J., Einhorn L., Kanz L. (2000). The relative risk of second nongerminal malignancies in patients with extragonadal germ cell tumors. Cancer.

[66] Nichols C.R., Hoffman R., Einhorn L.H., Williams S.D., Wheeler L.A., Garnick M.B. (1985). Hematologic malignancies associated with primary mediastinal germ cell tumors. Ann. Intern. Med..

[67] Mukherjee S., Ibrahimi S., John S., Adnan M.M., Scordino T., Khalil M.O., Cherry M. (2017). Non-seminomatous mediastinal germ cell tumor and acute megakaryoblastic leukemia. Ann. Hematol..

[68] Orazi A., Neiman R.S., Ulbright T.M., Heerema N.A., John K., Nichols C.R. (1993). Hematopoietic precursor cells within the yolk sac tumor component are the source of secondary hematopoietic malignancies in patients with mediastinal germ cell tumors. Cancer.

[69] Taylor J., Donoghue M.T., Ho C., Petrova-Drus K., Al-Ahmadie H.A., Funt S.A., Zhang Y., Aypar U., Rao P.N., Chavan S.S. (2020). Germ cell tumors and associated hematologic malignancies evolve from a common shared precursor. J. Clin. Investig..

[70] Chaganti R.S.K., Ladanyi M., Samaniego F., Offit K., Reuter V.E., Jhanwar S.C., Bosl G.J. (1989). Leukemic differentiation of a mediastinal germ cell tumor. Genes Chromosom. Cancer.

[71] Ladanyi M., Samaniego F., Reuter V.E., Motzer R.J., Jhanwar S.C., Bosl G.J., Chaganti R.S.K. (1990). Cytogenetic and Immunohistochemical Evidence for the Germ Cell Origin of a Subset of Acute Leukemias Associated With Mediastinal Germ Cell Tumors. J. Natl. Cancer Inst..

[72] Vlasveld L., Splinter T.A.W., Hagemeijer A., Van Lom K., Löwenberg B. (1994). Acute myeloid leukaemia with +i(12p) shortly after treatment of mediastinal germ cell tumour. Br. J. Haematol..

[73] Sowithayasakul P., Sinlapamongkolkul P., Treetipsatit J., Vathana N., Narkbunnam N., Sanpakit K., Buaboonnam J. (2018). Hematologic Malignancies Associated With Mediastinal Germ Cell Tumors: 10 Years’ Experience at Thailand’s National Pediatric Tertiary Referral Center. J. Pediatr. Hematol..

[74] Lu C., Riedell P., Miller C., Hagemann I.S., Westervelt P., Ozenberger B.A., O’Laughlin M., Magrini V., Demeter R.T., Duncavage E.J. (2016). A common founding clone withTP53andPTENmutations gives rise to a concurrent germ cell tumor and acute megakaryoblastic leukemia. Mol. Case Stud..

[75] Leonard J.T., Raess P.W., Dunlap J., Hayes-Lattin B., Tyner J.W., Traer E. (2016). Functional and genetic screening of acute myeloid leukemia associated with mediastinal germ cell tumor identifies MEK inhibitor as an active clinical agent. J. Hematol. Oncol..

[76] Oshrine B.R., Olsen M.N., Heneghan M., Wertheim G., Daber R., Wilmoth D.M., Biegel J.A., Pawel B., Aplenc R., King R.L. (2014). Acquired isochromosome 12p, somatic TP53 and PTEN mutations, and a germline ATM variant in an adolescent male with concurrent acute megakaryoblastic leukemia and mediastinal germ cell tumor. Cancer Genet..

[77] Akizuki K., Sekine M., Kogure Y., Kameda T., Shide K., Koya J., Kamiunten A., Kubuki Y., Tahira Y., Hidaka T. (2020). TP53 and PTEN mutations were shared in concurrent germ cell tumor and acute megakaryoblastic leukemia. BMC Cancer.

[78] Amra N., Zarate L.V., Punia J.N., Mahajan P., Stevens A.M., Roy A., Curry C.V., Cortes-Santiago N., Fisher K.E. (2020). Mediastinal Germ Cell Tumor and Acute Megakaryoblastic Leukemia With Co-occurring KRAS Mutation and Complex Cytogenetics. Pediatr. Dev. Pathol..

[79] Oosterhuis J.W., Looijenga L.H. (2020). Mediastinal germ cell tumors: Many questions and perhaps an answer. J. Clin. Investig..

[80] Nichols C.R., Heerema N.A., Palmer C., Loehrer P.J., Williams S.D., Einhorn L.H. (1987). Klinefelter’s syndrome associated with mediastinal germ cell neoplasms. J. Clin. Oncol..

[81] Levy D.R., Agaram N.P., Kao C.-S., Franks S.E., Kesler K.A., Stram A.R., Einhorn L.H., Bangs C.D., Ulbright T.M. (2020). Vasculogenic Mesenchymal Tumor: A Clinicopathologic and Molecular Study of 55 Cases of a Distinctive Neoplasm Originating From Mediastinal Yolk Sac Tumor and an Occasional Precursor to Angiosarcoma. Am. J. Surg. Pathol..

[82] Oing C., Kollmannsberger C., Oechsle K., Bokemeyer C. (2016). Investigational targeted therapies for the treatment of testicular germ cell tumors. Expert Opin. Investig. Drugs.

[83] Mego M., Svetlovska D., Chovanec M., Rečkova M., Rejlekova K., Obertova J., Palacka P., Sycova-Mila Z., De Giorgi U., Mardiak J. (2019). Phase II study of avelumab in multiple relapsed/refractory germ cell cancer. Investig. New Drugs.

[84] Adra N., Einhorn L.H., Althouse S.K., Ammakkanavar N.R., Musapatika D., Albany C., Vaughn D., Hanna N.H. (2018). Phase II trial of pembrolizumab in patients with platinum refractory germ-cell tumors: A hoosier cancer research network study GU14-206. Ann. Oncol..

[85] Shah S., Ward J.E., Bao R., Hall C.R., Brockstein B.E., Luke J.J. (2016). Clinical response of a patient to anti-PD-1 immunotherapy and the immune landscape of testicular germ cell tumors. Cancer Immunol. Res..

[86] de Bono J.S., Scher H.I., Montgomery R.B., Parker C., Miller M.C., Tissing H., Doyle G.V., Terstappen L.W., Pienta K.J., Raghavan D. (2008). Circulating tumor cells predict survival benefit from treatment in metastatic castration-resistant prostate. Clin. Cancer Res..

[87] Nicolazzo C., Busetto G.M., Gradilone A., Sperduti I., Del Giudice F., Loreni F., Cortesi E., De Berardinis E., Gazzaniga P., Raimondi C. (2019). Circulating Tumor Cells Identify Patients with Super-High-Risk Non-Muscle-Invasive Bladder Cancer: Updated Outcome Analysis of a Prospective Single-Center Trial. Oncologist.

[88] Nastaly P., Ruf C., Becker P., Bednarz-Knoll N., Stoupiec M., Kavsur R., Isbarn H., Matthies C., Wagner W., Höppner D. (2014). Circulating Tumor Cells in Patients with Testicular Germ Cell Tumors. Clin. Cancer Res..

